# Spatiotemporal Approaches to Assess the Association of Environmental Risk Factors With Cardiovascular Diseases: A Scoping Review

**DOI:** 10.1029/2024GH001268

**Published:** 2026-01-06

**Authors:** Vishal Singh, Susanna Cramb, Jialu Wang, Wenbiao Hu, Javier Cortes‐Ramirez

**Affiliations:** ^1^ School of Public Health and Social Work Queensland University of Technology Kelvin Grove QLD Australia; ^2^ Centre for Data Science Queensland University of Technology Kelvin Grove QLD Australia; ^3^ Australian Centre for Health Services Innovation Centre for Healthcare Transformation Queensland University of Technology Kelvin Grove QLD Australia

**Keywords:** spatial epidemiology, disease mapping, small‐area estimation, spatiotemporal data visualization, air pollution, temperature

## Abstract

Cardiovascular diseases (CVDs) remain a leading cause of mortality globally, with environmental risk factors playing a significant role in their prevalence. This review aims to critically evaluate the current methodologies employed in spatiotemporal analyses of CVDs and provides recommendations to enhance the accuracy and practical application of these models. A systematic search of the literature was conducted using Scopus, PubMed, and Embase databases. Studies were selected based on their use of spatiotemporal models to assess the relationship between environmental factors and CVDs. We evaluated the methodological quality of included studies using the Spatial Methodology Appraisal of Research Tool (SMART). Significant challenges were noted, including the need for higher spatial resolution data sets and improved methods for addressing the modifiable areal and temporal unit problems and ecological bias. Additionally, the visualization of spatiotemporal data remains underutilized and underdeveloped, limiting the practical utility of the findings. We also discuss combining parameters to form an indicator that better represents environmental conditions, as well as cases where ground, satellite, or modeled data products are suitable. These recommendations could extend to other acquired chronic diseases and their relationship with environmental risk factors to improve the utility of spatiotemporal models. While spatiotemporal modeling holds considerable promise in understanding and mitigating CVD risks associated with environmental factors, appropriate data selection, addressing methodological pitfalls and reporting spatial and temporal model outcomes are necessary to enhance their reliability and impact.

## Plain Language Summary

Cardiovascular diseases (CVDs), like heart attacks and strokes, are major causes of death worldwide, with environmental factors such as air pollution and temperature linked to their occurrence. The occurrence of CVDs and environmental factors are closely linked to the geographical location, as well as changes over time. This review looks at how researchers are using models that track changes over space and time to study these links. Our review highlights key challenges in these models, such as the need for more precise data on where people live and better methods to account for the way different time periods and regions are grouped. We also found that tools for visualizing this data are often underdeveloped, making it harder for researchers and policymakers to apply the findings in real‐world settings. We provide recommendations on choosing the best data sources to reflect environmental conditions accurately and combining several factors into one indicator to better represent environmental risks. These recommendations could improve the way we model and understand how CVDs and environmental factors are connected, benefiting research into other chronic diseases as well. By enhancing the data and methods used in these models, we can better understand and ultimately reduce CVD risks related to environmental factors.

## Key Points

- Spatiotemporal models provide a robust understanding of the relationship between health effects and environmental risk factors
- Current applications require improvements in spatial and temporal resolution of data sets to reduce generalization of exposure levels
- Advanced visualization tools are needed to interpret spatiotemporal data for improving their utility in a public health setting

## 1 Introduction

Cardiovascular diseases (CVDs) have remained a prominent cause of death worldwide (Vaduganathan et al., 2022). Even though CVD incidence has declined since the 1970s, this decrease has slowed or reversed in some high‐income countries (Lopez & Adair, 2019). Despite this, the global healthcare expenditure for CVDs is increasing, with current estimates to be around $1 trillion USD per year (Bloom et al., 2011). The CVD‐related deaths are disproportionately distributed across the world, where low‐ and middle‐income countries account for 75% of all CVD deaths, and some regions such as India report that 45% of deaths in the 40–69 age group are attributed to CVDs (World Health Organisation, 2024), resulting in large productivity costs. In 2020, CVDs accounted for 32.7% of all deaths in the European Union, significantly higher than cancer, the second leading cause of death at 22.5% (Eurostat, 2024).

It is estimated that environmental risk factors, including ambient and household air pollution, lead exposure and temperature extremes, are responsible for 19.4% and 18.6% of all CVD‐related deaths and disability‐adjusted life years, respectively (Vaduganathan et al., 2022). The age‐standardized years lived with disability rates attributed to air pollution have been on an upward trend. The environmental factors contributing to excess CVD risk, such as weather and pollution, vary over space due to geography, sources of pollution, urbanization patterns, land use characteristics and global climate systems dynamics. In addition, CVDs are also related to community‐level risk factors such as local dietary preferences and socioeconomic status, highlighting the complex interplay between location of residence and cardiovascular health outcomes. Given the disparities in CVD incidence and the dynamic nature of these factors, understanding the spatial and temporal characteristics in the relationship between CVDs and environmental risk factors is urgently needed.

The association of environmental risk factors with CVDs has traditionally been investigated through cohort, cross‐sectional and time‐series studies, typically overlooking location‐based information (Liu et al., 2023) and cannot identify place‐based health disparities. Location‐based analyses are therefore crucial to better understand these risks and formulate policies targeted at reducing health disparities. However, analyses of small‐area units in ecological studies can result in spurious results, especially when dealing with areas with low populations or high variability in incidence rates (Anderson & Ryan, 2017; Morris & Munasinghe, 1993). In using spatial statistical models, this issue can be minimized by incorporating a spatial structure for the relationship between each spatial unit (Sue Bell & Broemeling, 2000). This is possible under the assumption of the first law of geography, that areas closer to each other have similar characteristics than areas farther apart (Tobler, 1970). In contrast, some studies use models with only independent random effects, treating each area as a distinct and isolated spatial unit, which cannot be interpreted as a realistic assumption of spatial dependency (MacNab, 2022).

With environmental changes happening over space and time, the impact of these evolving conditions is important in epidemiological analysis as population characteristics and disease prevalence change over time. Exclusively spatial or temporal models do not allow the understanding of changes in risk for each spatial unit over time, as dynamic environmental conditions get aggregated over large areas or long time periods. In contrast, a spatiotemporal model can help draw strong inferences on both specific areas and time periods because of the inclusion of a spatial component assessed over time and a temporal component assessed across space (Blangiardo et al., 2013). Spatiotemporal models can also incorporate a space‐time interaction to better understand the overall and the area and time specific risk of a disease, and provide rich metrics to support decision making in public and environmental health (Cortes‐Ramirez, Wilches‐Vega et al., 2025).

Despite the appeal of spatiotemporal analyses in explaining epidemiological interactions, no review has focused on their use in examining the relationship between environmental risk factors and CVDs. To serve this purpose, this study aims to systematically search the extent of spatiotemporal analyses conducted for assessing the relationship between environmental risk factors and CVDs. To aid in future research and identify areas for improvement, this review will:

1. Examine the types of data sources and characteristics utilized for health and environmental variables.
2. Identify the types of statistical models used for estimating spatiotemporal exposure and effect estimates.
3. Examine visualizations used for the output of spatiotemporal models.

## 2 Methods

This review followed the Preferred Reporting Items for Systematic reviews and Meta‐Analyses guidelines for scoping review (PRISMA‐ScR) (Tricco et al., 2018). We developed our search strategy using the following eligibility criteria. For inclusion, studies should consider CVDs individually or as a group. Secondly, studies should assess at least one environmental risk factor (e.g., such as air pollution, temperature, or greenspaces), and thirdly, the studies should assess the relationship between environmental risk factors and CVDs through a spatiotemporal analysis, that is, the model considers spatial and temporal structures of both health and environmental data. Only peer‐reviewed original journal articles and letters published in the English language were considered without restricting the year of publication.

Based on our search strategy, we selected three search databases, Scopus, PubMed, and Embase and conducted the search on 23 January 2024 without restrictions on year of publication. For Scopus and Embase, title, abstract and keywords were searched, while MeSH terms were searched instead of keywords in PubMed. The keywords included terms related to CVDs, such as “cardiovascular,” “heart disease,” “cerebrovascular,” “stroke” and “cardiorespiratory”; environmental factors, such as “environment*,” “air quality,” “pollutant,” “temperature,” “green space*”; and spatiotemporal models such as “spatial,” “geospatial” and “spatio‐temporal.” More information regarding search terms used in each database can be found in Appendix A.

The screening process was conducted manually using EndNote. After removing duplicate records, the titles and abstracts screening and full‐text screening were performed by two authors, and disagreements were resolved through discussion between three authors. The data extraction was performed manually and entered in Microsoft Excel. Extracted data included first author, year of publication, study aim and design, study location and period of health data, population characteristics, data source and spatial and temporal extent and resolution, statistics employed in getting exposure measurements, spatiotemporal models, including characteristics of spatial, temporal and interaction terms, and information on how the outcomes were reported.

To assess the methodological quality of the selected studies, we used the Spatial Methodology Appraisal of Research Tool (SMART), a recently developed and validated framework tailored to spatial epidemiological research (Wood et al., 2025). Unlike other quality appraisal instruments, SMART explicitly addresses issues such as spatial scale, resolution, and spatial dependence, which are critical to evaluating spatial analyses. The SMART assesses four domains: methods preliminaries, data quality, spatial data problems, and spatial analysis methods. Each item within these domains is evaluated using categorical responses: yes, no, or other (e.g., cannot determine, not applicable, and not reported). Two reviewers (VS and JW) conducted the appraisals independently, resolving disagreements by consensus.

## 3 Results

### 3.1 Selection of Sources of Evidence

**Figure 1:**
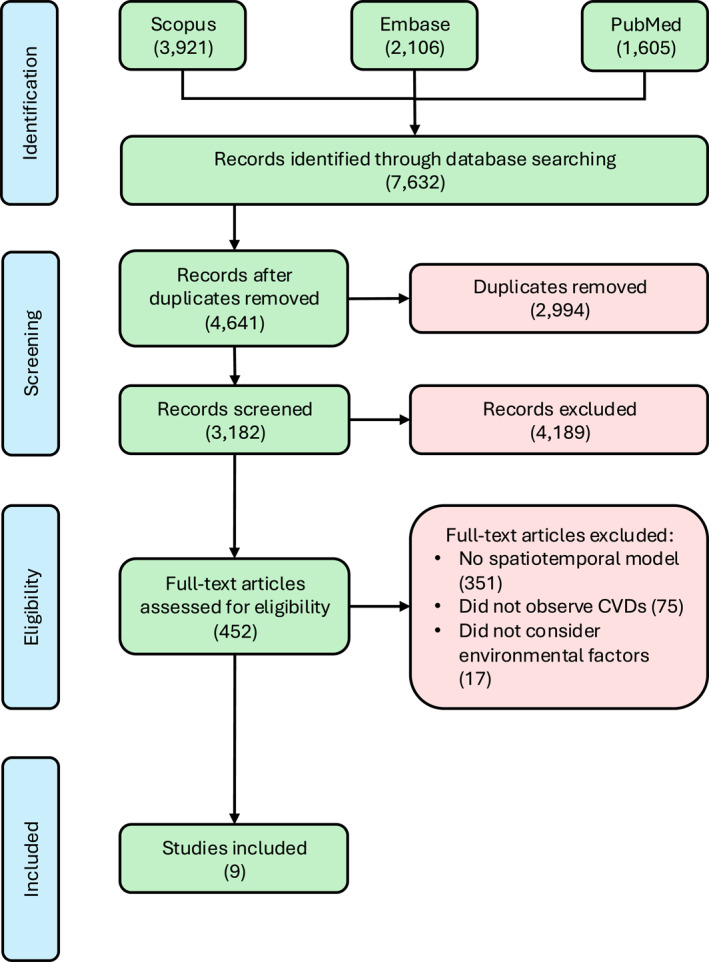
Screening process using the PRISMA flow diagram (Page et al., 2021).

**Figure 2:**
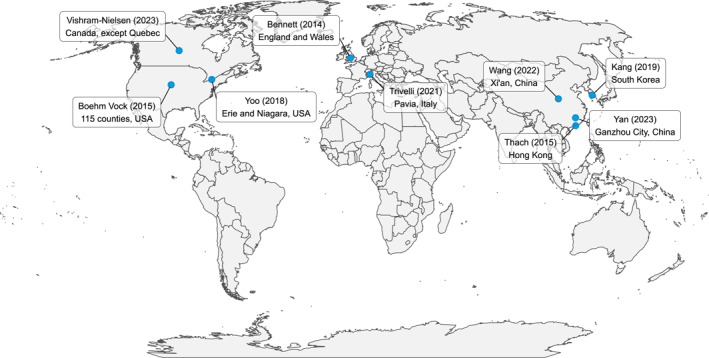
Study locations with first author and year of publication.

A total of 7,632 references were obtained through a systematic search in the selected databases, resulting in 4,641 records after removing duplicates. Subsequently, 1,544 studies were excluded based on predefined exclusion criteria. Following the title and abstract screening, 452 studies were selected for full‐text screening, of which 9 were deemed eligible for inclusion. A large number of studies (351) that did not pass the full‐text screening process were excluded because either they were not spatiotemporal models or they did not consider the spatial dependency in their modeling approach, so they were not considered spatiotemporal models for the purpose of this review (Liss & Naumova, 2019; Xu et al., 2014). The selection flowchart is shown in Figure 1, and a more compressive flowchart distinguishing the proportion of studies going through each step is available in the Figure S1 of Supporting Information S1. All included studies were published after 2014 and originated from eight countries (Canada, China, England, Wales, Hong Kong, Italy, South Korea, and the United States). The studies location and year of publication are presented in Figure 2.

### 3.2 Characteristics of Studies

#### 3.2.1 Health Data

The health data used in the selected studies was primarily sourced from governmental agencies, with the exception of Yan et al. (2023) that used hospital data. Six studies assessed CVDs as a collective group (Bennett et al., 2014; Boehm Vock et al., 2015; Kang et al., 2019; Thach et al., 2015; Trivelli et al., 2021; Yan et al., 2023), while three examined specific subcategories within CVDs (Vishram‐Nielsen et al., 2023; Wang et al., 2022; Yoo et al., 2018). Cardiovascular‐specific mortality was the health outcome in five studies, while two studies assessed emergency department visits (Boehm Vock et al., 2015; Yoo et al., 2018) and another two studies assessed hospital admissions(Vishram‐Nielsen et al., 2023; Yan et al., 2023).

**Figure 3:**
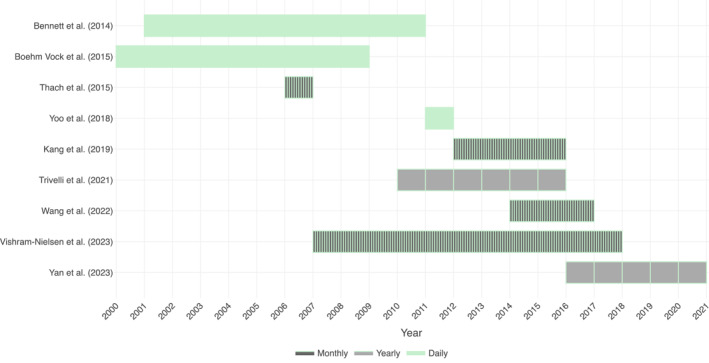
Study temporal scope and resolution with author and year of publication.

**Figure 4:**
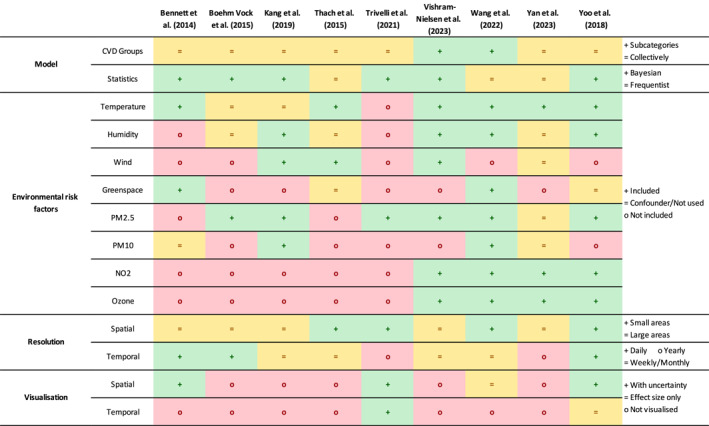
Summary of results.

The spatial resolution of the available health data ranged from precise (home addresses), small (e.g., municipality level) and large (e.g., district level). For the purposes of this review, spatial resolution was considered small if the average area per spatial unit is less than 100 km^2^. Four studies analyzed the health data at small‐area level (Thach et al., 2015; Trivelli et al., 2021; Wang et al., 2022; Yoo et al., 2018), with all other studies using larger areas in their analysis. Health records from 2000 to 2020 were assessed across the selected studies, with the temporal span ranging from one to 10 years (Figure 3). Daily health records were available in all except the Kang et al. (2019) study (monthly health data) and the Trivelli et al. (2021) study (yearly health data). However, four studies used monthly and yearly aggregated data while having daily temporal resolution health data (Thach et al., 2015; Vishram‐Nielsen et al., 2023; Wang et al., 2022; Yan et al., 2023). A summary of results is shown in Figure 4 and description of the studies can be found in Table 1. Additional information is available in Table S1 of Supporting Information S1.

**Table 1:** Description of Health and Environmental Data Used in Each Study

Author and year	Health data			Environmental data				
	Study population and size	Study location and spatial resolution	Temporal scope and resolution	Weather	Air pollutants	Other factors	Spatial resolution	Temporal resolution
Bennett et al. (2014)	Cardio‐respiratory diseases related deaths (over 75 years of age); *[Population size not stated]* 921,288 deaths	England and Wales; all 376 districts (large units) *[data available at post code level, ST modeling at district level]*	2001–2010; daily (3,652 days)	Temperature: Mean daily temperature, calculated as the average of daily maximum and minimum temperatures Used the average of temperature on day of death and the preceding 3 days to allow for the possibility that the health effects may be cumulative	*PM_10_: as a confounding factor* *Used an average of PM_10_ on the day of death and on preceding 2 days*	*Green space: as a modifier. As the percentage of the Census ward*	5 km × 5 km	Daily
Boehm Vock et al. (2015)	Cardiovascular emergency room admissions among Medicare patients; *[Population size or number of cases not stated]*	USA; 115 counties (large units)	2000–2008; daily (3,287 days)	*Temperature: As a confounding factor* *Dewpoint: As a confounding factor*	PM_2.5_: 22 components of PM_2.5_ measured separately		Monitoring stations	Daily
Kang et al. (2019)	CVD‐related deaths; *[Population size or number of cases not stated]*	South Korea; all 250 districts (large units)	2012–2015 (2012–2014 for model fitting, and data from 2015 for forecasting and evaluation); monthly (36–48 months)	*Temperature: As a confounding factor* *Humidity: As a confounding factor. [Assumed to be relative humidity]* *Wind speed: As a confounding factor*	PM_2.5_ *PM_10_*		Monitoring stations	Monthly
Thach et al. (2015)	CVD‐related deaths; 6.8 million population *[total number of deaths not stated]*	Hong Kong (Special administrative region, China); 276 Tertiary Planning Units (small units)	2006; monthly (12 months) *[data available at daily resolution, ST modeling at monthly resolution]*	*Temperature: Used to form PET values* *Humidity: Used to form PET values. Assumed to be relative humidity*. *Cloud cover: Used to form PET values* *Wind velocity: Used to form PET values*		*UC‐AnMap data comprising of meteorological, planning, land use, topography, and greenery information: used to form PET values* *Percentage of greenery and buildings: as a confounding factor*	100 m × 100 m grid	*[Temporal resolution for UC‐AnMap data is not stated, presumed to be constant* *Temporal resolution for data from Hong Kong Observatory is also not stated, presumed daily, which was aggregated to monthly.]* The final PET values were monthly for the GAM model.
Trivelli et al. (2021)	CVD‐related deaths; 535,666 population in 2012 (14,183 CVD‐related deaths)	Province of Pavia, Italy; 188 municipalities (small units)	2010–2015; yearly (6 years)		PM_2.5_		0.1° *[approximately 11 km × 8 km near study region]*	Annual mean
Vishram‐Nielsen et al. (2023)	Hospital admissions related to heart failure, MI, hemorrhagic and ischemic strokes, and ventricular and supraventricular tachycardia; 24 million population (1.7 million deaths)	Canada, except Quebec Province; 100 aggregated regions (large units) *[data available at home address level, ST modeling after aggregation to larger regions]*	2007–2017 (2007–2016 for model fitting, and data from 2017 for prediction evaluation); weekly (520 weeks) *[data assumed to be available at daily resolution, ST modeling at weekly resolution]*	Temperature: 2 m temperature, diurnal temperature change, and rate of diurnal temperature change Boundary layer height Evaporation Instantaneous eastward turbulent surface stress Instantaneous northern turbulent surface stress Surface pressure Volumetric soil water layer 1 Total cloud cover Total precipitation 10 m U wind component 10 m V wind component	PM_2.5_ O_3_ NO_2_ SO_2_		1.5° *[approximately 167 km × 123 km to 167 km × 82 km near the study region]*	12‐hourly (aggregated to daily values using CDO software), except for diurnal temperature and pressure that was calculated from hourly data.
Wang et al. (2022)	Acute MI deaths; 12.95 million population in 2020 (17,888 acute MI deaths between 2014 and 2016)	Xi'an, Shaanxi Province, China; 178 townships (small units) *[data available at home address level, ST modeling at township scale]*	2014–2016; monthly (36 months) [data available at daily resolution*, ST modeling at monthly resolution*]	Temperature Humidity: relative humidity	PM_2.5_ PM_10_ O_3_ NO_2_ NO_x_ SO_2_ CO Black carbon Organic carbon Ammonia	Digital Elevation Model *Environmental conditions considered as socioeconomic indicators:* Polluting enterprises Nightlight image NDVI	Monitoring stations: hourly CO, NO_2_, O_3_, PM_10_, PM_2.5_ and SO_2_, daily temperature, relative humidity, precipitation, pressure, and wind speed 30 m × 30 m: Digital Elevation Model Spatial resolution not stated: monthly Black Carbon, CO, CO_2_, Ammonia (NH_3_), NO_x_, organic carbon, SO_2_, volatile organic compounds, PM_2.5_ and PM_coarse_	Hourly: CO, NO_2_, O_3_, PM_10_, PM_2.5_ and SO_2_ Daily: temperature, relative humidity, precipitation, pressure, and wind speed Monthly: Black Carbon, CO, CO_2_, Ammonia (NH_3_), NO_x_, organic carbon, SO_2_, volatile organic compounds, PM_2.5_, PM_coarse_, nightlight image, and NDVI Static: polluting enterprises
Yan et al. (2023)	CVD‐related hospitalizations among people aged 25–85 years who resided more than 5 years in Ganzhou city; *[Population size or number of cases not stated]*	Ganzhou City, Jiangxi Province, China; 18 counties (large units) *[data available at home address level, ST modeling at county level]*	2015–2020; yearly (6 years) *[data available at daily resolution, ST modeling at yearly resolution]*	Temperature Temperature difference *Air pressure: Not used* *Wind speed: Not used* *Relative humidity: Not used* *Precipitation: Not used* *Sunshine hours: Not used*	O_3_ NO_2_ *PM_2.5_* *: Not used* *PM_10_: Not used* *SO_2_: Not used*		Environmental data was available for each spatial unit from source	Yearly
Yoo et al. (2018)	CVD‐related emergency department visits of the elderly (>65 years); 1,135,474 population (5,798 patients)	Buffalo‐Niagara region within Erie and Niagara counties of western New York, USA; ∼75 zip codes *[number unspecified]* (small units)	2011; daily (365 days)	*Temperature: Average temperature, and apparent temperature: not used* *Humidity: relative humidity and dew point temperature: not used*	PM_2.5_ O_3_ NO_2_ *SO_2_: Not used*	*Percentage of park area designated as state, county, or municipal parks per Zip: as a confounding factor*	Monitoring stations: Meteorological data Census tract: PM_2.5_ and ozone 12 km × 12 km: NO_2_	Daily

*Note.* Italicized text provides additional information about the data, including: lack of information (e.g., population size not stated), variation in the resolution of original data and the data used in ST modeling (e.g., data at postcode level and modeling at district level), parameters that were not included in the ST model (e.g., data on temperature was obtained but not used in the final model or used as a confounding factor), and parameters that were combined to form indicators that were used in the ST model (e.g., temperature and humidity used to form PET values). Acronyms: CDO, Climate Data Operators; GAM, Generalized Additive Model; MI, Myocardial Infarction; PET, Physiologically equivalent temperature; UC‐AnMap, Urban Climatic Analysis Map.

#### 3.2.2 Environmental Risk Factors

The sources for environmental risk factors data varied, and none of the studies used the same data sets. The most common environmental risk factors considered were temperature (8 studies), PM_2.5_ (7 studies), humidity‐related indicators (7 studies), and four studies each for greenspace‐related indicators, wind, PM_10_, NO_2_ and ozone (Table 1, Figure 4). The spatial resolution of the data ranged from 30 m squared (Wang et al., 2022) to about 167 km × 123 km (Vishram‐Nielsen et al., 2023), while the temporal resolution ranged from hours to years. Additional details are in Table S1 of Supporting Information S1.

Multiple indicators were used for assessing humidity, including dew point (Boehm Vock et al., 2015; Yoo et al., 2018), precipitation, evaporation and volumetric soil water layer (Vishram‐Nielsen et al., 2023), and relative humidity (Kang et al., 2019; Thach et al., 2015; Wang et al., 2022; Yan et al., 2023). Greenspace was measured as percentage of greenery (Thach et al., 2015), percentage of park area (Yoo et al., 2018), monthly normalized difference vegetation index (Wang et al., 2022) and percentage of green space (Bennett et al., 2014). Wind and atmospheric pressure were used in the Vishram‐Nielsen et al. (2023) study while Thach et al. (2015) used wind speed to form the physiologically equivalent temperature (PET), and Kang et al. (2019) used wind speed.

### 3.3 Spatiotemporal Models

A Bayesian modeling approach was used in all studies, except for three that used a frequentist approach (Thach et al., 2015; Wang et al., 2022; Yan et al., 2023; Table 2). The neighborhood around a spatial unit was considered by either using an adjacency matrix (Bennett et al., 2014; Kang et al., 2019; Trivelli et al., 2021; Vishram‐Nielsen et al., 2023; Wang et al., 2022; Yan et al., 2023; Yoo et al., 2018), a variable neighborhood selection based on distance (Boehm Vock et al., 2015), or spline smoothers considering geographic coordinates (Thach et al., 2015). Statistically significant and credible associations were found between environmental risk factors and CVDs; both cold and warm temperature extremes and all air pollutants were detrimental to CVD outcomes. Additional details on the statistical models and outcomes are in Table S1 of Supporting Information S1.

**Table 2:** Summary of Spatiotemporal Models and Outcome Reporting of Each Study

Author and year	Spatiotemporal models				Outcome reporting		
	Spatiotemporal statistics summary	Space	Time	Space‐time^a^	Spatial	Temporal	Fixed effect/other
Bennett et al. (2014)	Time‐stratified case‐crossover design with a piecewise linear model, setting district specific temperature thresholds using DIC values. Seasonal differences were assessed by stratifying months	BYM CAR	AR(2)	–	Map of different age groups and sex combinations of 1°C increase and decrease in temperature from district‐specific thresholds or national thresholds. Map of posterior probabilities is also reported	No	Summary table (odds ratio for overall effect for each age group and gender combination for 1°C increase or decrease in warm or winter season respectively)
Boehm Vock et al. (2015)	Three Bayesian spatial models: spatial model without VS, exchangeable VS model, and spatial VS model. Different natural cubic spline smoothing functions of time for different variables are used, and three types of SC (no SC, moderate SC, and strong SC)	Spatial variable selection	Cubic spline	–	No	No	Reported in text
Kang et al. (2019)	Two stage Bayesian hierarchical spatiotemporal model, where residuals are obtained from a covariates‐only model and these residuals are used for fitting a space‐time mixture structure to estimate locally different temporal components. They used two parallel MCMC with different initial values and evaluated the models using DIC and MSPE	CAR	AR(1)	Type III	No	No	Summary table (overall relative risk for PM_2.5_ and PM_10_)
Thach et al. (2015)	GAM model assuming a Poisson distribution for count data. Seasonal differences were assessed by stratifying months	Tensor product spline	Cubic spline	–	No	No	Summary table (excess risk for warm and cold season before and after adjusting for socio‐economic deprivation index)
Trivelli et al. (2021)	Bayesian hierarchical models using Hamiltonian MCMC approach. The modeling was done in four phases with and without temporal parametric trends and the best model from each phase informed the prior for the next phase of the analysis. All the phases were Poisson log‐linear models with increasing complexity where models 3B and 4B were the only spatiotemporal models with 3B including only spatially structured effects and model 4B including spatially structured and unstructured effects	CAR	Temporal trend	–	Maps of relative risk for each year. Map of posterior probabilities is also reported. Map of overall risk not reported	Maps of relative risk for each year. Overall trend of relative risk not available	Summary table (overall posterior distributions for each PM_2.5_)
Vishram‐Nielsen et al. (2023)	BYM model specification for spatial terms and linear time effect and dynamic nonparametric trend for temporal effect with an interaction term of type I were computed using INLA. The independent variables and variable pairs underwent a selection process by WAIC comparison	BYM CAR	RW(1)	Type I	No	No	Bubble chart (probability of risk being smaller or greater than 1; strength of association not obtainable through the bubble chart and not reported in text)
Wang et al. (2022)	GTWR that incorporated the location and time of each variable included	GTWR	GTWR	–	Map of all environmental risk factors at different lags for all genders. Map of model residual is also reported	No	Summary table (all lags and genders for all variables)
Yan et al. (2023)	Spatial panel Durbin model with time‐lagged dependent variable and space‐time lagged dependent variable using fixed time effects and spatial random effects	Not sure	Not sure	–	No	No	Summary table (overall risk for each independent variable)
Yoo et al. (2018)	Bayesian hierarchical Poisson linear model with a BYM spatial structure using INLA for estimation. The temporal variable included a within‐year cyclical pattern, day of the week and holidays, and a linear trend	CAR	Temporal trend	–	Map of overall risk. Map of exceedance probability is also reported. Map of relative risk for the individual factors is not reported	Longitudinal line chart of overall risk	Summary table (relative risk of all variables with 95% confidence interval and standard deviation)

*Note.* Acronyms: AR, AutoRegressive; BYM, Besag‐York‐Mollie; CAR, Conditional AutoRegressive; DIC, Deviance Information Criteria; GAM, Generalized Additive Mixed; GTWR, Geographically and Temporally Weighted Regression; MCMC, Monte Carlo and Markov Chains; MSPE, Mean Squared Prediction Error; RW, Random Walk; SC, Spatial Correlation; VS, Variable Selection.

^a^ Space‐time interaction terms classified as defined by Knorr‐Held (2000), where Type I is when unstructured space and time terms interact (all observations are independent), Type II is when unstructured space and structured time terms interact (each spatial unit follows its own path in time), Type III is when structured space and unstructured time terms interact (spatial units interact with each other but differ in time), and Type IV is when structured space and time terms interact (spatial units interact with each other and in time).

### 3.4 Outcome Reporting and Visualization

All studies provided the overall fixed effect of the change in CVDs in relation to the variable relevant to their objectives, except Vishram‐Nielsen et al. (2023) that reported results as either positive or negative associations (Table 2). Spatial unit‐specific effect estimates were visualized as overall spatial random effects by Bennett et al. (2014) and Yoo et al. (2018), for each time unit by Trivelli et al. (2021), and for each environmental risk factor and model residuals at different lags by Wang et al. (2022) (Figure 4). The remaining five studies did not present spatial random effects. Most studies did not provide temporal random effect estimates, except for Yoo et al. (2018) who provided a longitudinal line chart of overall relative risk across years without confidence intervals, and Trivelli et al. (2021) who provided a map of relative risk for each year. Only three studies provided posterior probabilities that could be used to draw inferences regarding the plausibility that the estimates differ from the average (Bennett et al., 2014; Trivelli et al., 2021; Yoo et al., 2018).

### 3.5 Quality Appraisal

**Figure 5:**
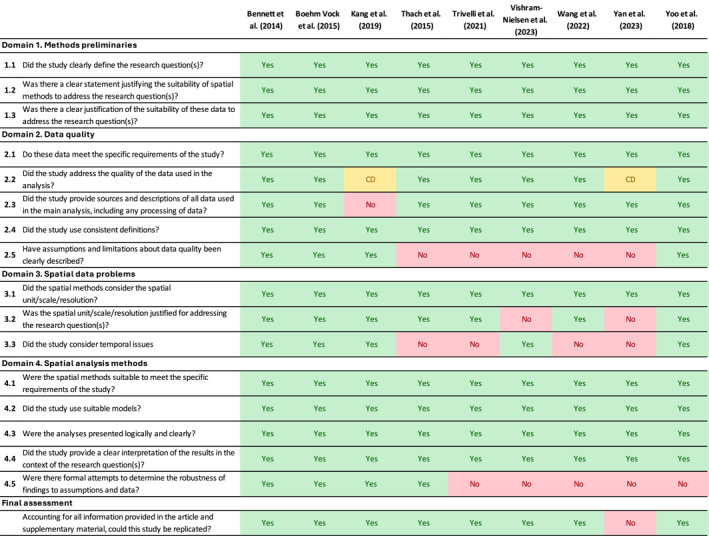
Quality appraisal of included studies using Spatial Methodology Appraisal of Research Tool (SMART). *CD, Cannot Determine; “No” encompasses “Not Reported” rating of the SMART Tool.

Across the nine studies assessed with the SMART, all defined the research question, justified the use of spatial methods, and selected suitable models with results presented and interpreted appropriately (Figure 5). The data suitability was addressed in all but three studies using data that were not suitable for the analysis (Trivelli et al., 2021; Vishram‐Nielsen et al., 2023; Yan et al., 2023). Reporting on data quality and limitations was less consistent. Kang et al. (2019) did not specify the source of environmental data, and five studies (Thach et al., 2015; Trivelli et al., 2021; Vishram‐Nielsen et al., 2023; Wang et al., 2022; Yan et al., 2023) did not adequately discuss data limitations. All studies reported information on spatial units and resolution, except for two studies that provided insufficient justification for their choices (Vishram‐Nielsen et al., 2023; Yan et al., 2023). Temporal issues were more variably addressed, with four studies aggregating or handling time without adequate justification (Thach et al., 2015; Trivelli et al., 2021; Wang et al., 2022; Yan et al., 2023). Only four studies (Bennett et al., 2014; Boehm Vock et al., 2015; Kang et al., 2019; Thach et al., 2015) performed sensitivity analyses to test robustness. Replicability was possible for most studies, except Yan et al. (2023) which lacked sufficient methodological detail. Overall, the quality appraisal showed good use of spatial methods but highlighted weaknesses in addressing data quality, temporal justification, and sensitivity analysis. Additional information on the rationale for each rating is presented in Table S2 of Supporting Information S1.

## 4 Discussion

This is the first review of studies that used spatiotemporal analyses to examine the relationship between environmental risk factors and cardiovascular diseases. We used the PRISMA‐ScR framework rigorously to identify nine studies that used joint spatiotemporal analyses for assessing this relationship. These studies varied regarding the spatial and temporal extent and resolution of their health and environmental data sets, using Bayesian and frequentist approaches and temperature and PM_2.5_ as the most common environmental risk factors. All the included studies found statistically significant and credible associations between CVDs and environmental risk factors and showed spatial dependence and temporal evolution of this relationship when disclosed in the study. While the studies pursued diverse objectives shaping their reporting approach, all could have used additional comprehensive spatial and temporal data visualizations. The studies were assessed with the Spatial Methodology Appraisal of Research Tool (SMART), which identified issues related to inadequate justification for data quality, temporal aggregation and lack of sensitivity analyses.

### 4.1 Relationship Between Environmental Risk Factors and Cardiovascular Diseases

Our findings show that the spatiotemporal relationship between CVDs and PM_2.5_, PM_10_, NO_2_ and O_3_ is either positively associated or not statistically credible or significant. This aligns with previous environmental health epidemiological studies and can be explained by well‐established causal links (Al‐Kindi et al., 2020; Jiang et al., 2023; Orellano et al., 2020; Stieb et al., 2020). CVDs are a large group of diseases that can be affected by diverse environmental factors with distinct causal pathways leading to various cardiovascular health outcomes. These include many CVDs linked to the development of atherosclerotic plaques facilitated by chronic exposure to air pollution and other individual‐level stressors (Bevan et al., 2021). This poses a higher risk of acute CVD events through vascular flow disturbances mediated by air pollution, temperature deviations or other environmental factors.

The relationship between various types of CVDs and environmental risk factors may differ, including the strength of the association, time of onset, or interactive effects with other risk factors such as diabetes comorbidity (Xu et al., 2022). Cold temperatures can have short (hours) to intermediate‐term (weeks) CVD risk, whereas the effects of high temperature on the cardiovascular system are short‐term (days) and impermanent (Chen et al., 2022; Liu et al., 2022). However, the studies included in this review mostly analyzed CVDs collectively rather than specific sub classifications. Specific CVDs, such as arrhythmia and hypertension, have different mechanisms and time lags related to air pollution and temperature, which should be considered when exploring these relationships in epidemiological studies (Chen et al., 2022; Goel et al., 2022; Qin et al., 2021; Zhang et al., 2021). Therefore, there is benefit in analyzing cardiovascular diseases separately for each risk factor with emphasis on the differences in the mechanism of the temperature variation effects. Furthermore, temperature can be analyzed with various metrics such as mean, minimum or maximum daily temperature, diurnal temperature change within a day, monthly averages, or heatwaves. However, temperature alone is not a good measure of thermal comfort as it depends on multiple other factors, including humidity, wind speed, solar radiative heat, clothing, etc (Havenith & Fiala, 2015). For this purpose, numerous heat indices have been created, and the most used include apparent temperature and wet bulb globe temperature (Bureau of Meteorology, 2010). Although these indices better represent “feels‐like” temperature, they are rarely used—only one selected study used physiologically equivalent temperature (Thach et al., 2015). Hence, more research is needed on the relationship between CVDs and various temperature indicators, and multiple indices used in the same population can help elucidate the differences observed in the outcome.

As the effects of most environmental factors considered in the studies in this review can have immediate or short‐term effects on cardiovascular health, metrics of their temporal aggregation can be especially important for health risk estimation. Monthly or yearly temporal resolution can underestimate the health risk of the short‐term changes in environmental conditions by averaging over a long period of time. More than half of the studies included in this review used monthly or yearly aggregated data. Our findings show that the CVD risk associated with temperature is less pronounced when the analysis is done on yearly aggregated data (Yan et al., 2023) as both hot and cold temperature deviations can result in poor CVD outcomes (Bennett et al., 2014; Thach et al., 2015). This can be due to the non‐monotonic relationship of CVDs with temperature (Wen et al., 2023; Wu et al., 2023; Zhai et al., 2022). Thus, yearly temporal aggregation can be unsuitable when investigating the effect of temperature and other dynamic environmental conditions on CVD health outcomes. This variability underscores the importance of assessing both the spatial and temporal dimensions of data used in public health research. A better understanding of this relationship can inform the development of targeted preventive or mitigation strategies that account for local evolving conditions and support prediction modeling for various environmental scenarios.

We found that Bayesian modeling approaches were more common, possibly due to their ability to include multiple data sources and prediction of uncertainty for specific areas, allowing more flexible and extensive spatiotemporal models compared with the frequentist approach (Lawson et al., 2016). Hierarchical specifications are well‐suited to use the Bayesian approach to allow the inclusion of prior distributions to inform evidence built up on previous research on more frequent and severe environmental exposures such as wildfires. For Bayesian models, novel methods for parameterization of inference, such as the Integrated Nested Laplace Approximation (INLA), offer an alternative to traditional and computationally expensive Markov‐Chain Monte Carlo (MCMC) methods, especially when large spatiotemporal data sets are involved (Rue et al., 2009). Furthermore, refinements to MCMC and INLA approaches in improving their efficiency, combined with increasing processing capabilities of modern computers, are making spatiotemporal analysis more approachable (Wang et al., 2024).

### 4.2 Data

Despite the utility of spatiotemporal analyses, the small number of studies identified in this review highlights that their adoption in epidemiological research on CVDs has been limited. This can be partly attributed to challenges related to the data. Firstly, acquiring health data with information on location and time can lead to patient identifiability concerns, which can limit the provision of data for maintaining confidentiality. Additionally, the data on various environmental factors are often available in different formats with differing spatial and temporal resolutions, making the selection of data sets and preparation for analysis very resource intensive.

#### 4.2.1 Health Data

Spatially aggregated data analyses follow an ecological design which introduces potential ecological bias where the inferences drawn at the population level may not represent the relationships at the individual level (Haining et al., 2010). Spatiotemporal analyses can help control ecological bias through methodological choices, such as adapting model specification per data distribution (Lawson et al., 2016), uniformly standardizing dependent and independent variables (Greenland & Robins, 1994), adjusting for confounding factors (Morgenstern, 1995), and using hierarchical mixed models (Cortes‐Ramirez, Mengersen, et al., 2024; Richardson et al., 2004). Spatial analyses can also help to reduce the impact of small counts of events in very large areas and the opposite effect of large counts in small, usually urban, geographical areas by smoothing estimates across all spatial units to provide more robust estimates (Blangiardo et al., 2013). Data aggregation can adversely affect the analysis outcome in terms of explaining local variations and statistical power, with higher‐resolution data outperforming low‐resolution data (Hanigan et al., 2017). The stability of rates is linked to the resolution of data; for instance, places with lower population at risk in smaller areal aggregate units can show spurious rates, also known as the small number problem, which should be investigated for its impact on the outcome through aggregation to different scales (Kok et al., 2021; Nelson & Brewer, 2017). The aggregation of data can also lead to issues such as the Modifiable Areal Unit Problem (MAUP), where the characteristics of spatial units, like size, shape, and orientation, affect the outcome of the analysis (Openshaw, 1984). Similarly, the Modifiable Temporal Unit Problem (MTUP) occurs when the aggregation, segmentation, and boundaries of time periods influence outcomes (Cheng & Adepeju, 2014). These challenges can be addressed when aggregation is minimally introduced, that is using small areal and temporal units, because the aggregation will be more sensitive to the underlying data (Hanigan et al., 2017; Kok et al., 2021). Using high spatial and temporal resolution in the data can also enable targeted policy intervention strategies with efficient and fair resource allocation (Hanigan et al., 2017; Tessema et al., 2023).

#### 4.2.2 Environmental Data

Our findings show that spatiotemporal analyses of CVDs use environmental data from either monitoring stations or modeled by environmental agencies. For example, one of the selected studies used a was 1.5° spatial resolution (Vishram‐Nielsen et al. (2023), which might not be ideal for studying health effects as the exposure levels are being averaged over a large area, resulting in poor estimation of exposure for many communities (Ranjan et al., 2021). However, access to high‐resolution, accurate and reliable environmental data can be a major challenge in spatial epidemiology. Air pollutants and meteorological variables can be accurately measured using ground‐based monitoring equipment, but they may be sparsely or unevenly distributed, resulting in unreliable estimates farther from the stations due to varying land cover types, geographical attributes and weather patterns (Tian et al., 2023). Satellite data products can be used, but they are inherently less accurate than in situ measurements. Polar synchronous satellites provide better spatial coverage but suffer from low temporal resolution, while geosynchronous satellites have higher temporal resolution but lower spatial resolution and do not have global coverage (Zhu et al., 2023).

Integrating ground and satellite‐based data sets can potentially provide better spatial and temporal coverage and resolution with higher accuracy and reliability for regions with sparsely distributed monitoring stations (Bai et al., 2023). Nevertheless, the availability of data products with these attributes with global coverage is limited (Chu et al., 2016; Ranjan et al., 2021). Data from monitoring stations can be used if they are densely placed in a manner that covers the population of interest; otherwise, estimates can be modeled through various modeling techniques (Ma et al., 2024; Tian et al., 2023). New data sets are being released with improved accuracy, but it is challenging to recommend a single data set as some may have limited temporal coverage (Wei et al., 2023), spatial resolution (Hersbach et al., 2023; Yu et al., 2023), or other aspects that could make them unsuitable for a specific study design. There is a need for more open‐access data sets that have high spatial (≤1 km) and temporal (≤1 day) resolution, global coverage with long‐term historical records and are updated frequently. These data sets should be accurate across various exposure levels, land cover types and geographical variations and validated across different regions worldwide. Exposure representativeness at small area levels will improve as the resolution and accuracy of environmental data increase (Ranjan et al., 2021).

### 4.3 Visualizing Model Outputs

Spatiotemporal analyses can produce a rich set of results, further exploring the spatiality and temporality of the modeled relationship and visualizing them using maps or time charts to enhance the communication of findings (Cortes‐Ramirez, Singh, et al., 2025; Cortes‐Ramirez, Wilches‐Vega et al., 2024). These make complex data more accessible and understandable facilitating the translation of research into actionable insights for local and broader policy‐level changes (Haining, 2003). However, the use of rich data visualizations was not a common practice among the selected studies which compounded with the lack of reporting of estimate uncertainty in maps and longitudinal line charts. Expanding the use of spatial and temporal visualizations in future research can increase the impact of complex data analyses for practical applications. This ensures that findings are not only scientifically robust but also easily interpretable for policymakers and public health professionals. Although spatiotemporal models can generate a wealth of outputs beyond summary tables of fixed effects, presenting these outputs within the confines of journal articles and reports can be intractable. Interactive web applications represent a promising solution for providing extensive details (Cancer Council Queensland and Queensland University of Technology, 2024). These applications can effectively communicate results in a thorough and accessible manner, enhancing the understanding of complex outputs. By allowing users to interact with the data, explore various scenarios, and visualize spatial and temporal patterns dynamically, web applications can significantly improve the translation of research findings into actionable insights. This approach not only makes the data more engaging but also facilitates more informed decision‐making at both local and broader policy levels.

### 4.4 Limitations

Although we rigorously implemented the PRISMA‐ScR framework, there are some limitations. The search strategy aimed to include all relevant peer‐reviewed research, but some relevant studies may not have been indexed in the three databases used or may not have been written in the English language. Excluding studies published in other languages could have introduced selection bias by not including studies done in non‐English speaking regions. The selection criteria implied that only direct measurements or indicators of environmental factors were included; therefore, proxies, such as the presence of potential polluting sources without determined exposure levels, were excluded. By excluding proxy indicators, we systematically reduced measurement bias from our review, given that exposure estimations rely heavily on assumptions made by the authors, which may not be verifiable given the lack of measurements.

## 5 Conclusion

This review provides a comprehensive overview of the current landscape of spatiotemporal analyses linking cardiovascular diseases to environmental risk factors. Consistent associations between CVDs and air pollutants like PM_2.5_ and temperature were identified. The findings affirm the importance of considering spatial and temporal dimensions conjointly in epidemiological studies. However, the availability of health and environmental data sets fit for small‐area modeling remains challenging. Despite the methodological advancements in spatiotemporal models, significant gaps remain in reporting the results of the random effects, especially regarding their uncertainty.

To maximize the impact of future research, there is a clear need for addressing these limitations through consideration of modifiable areal and temporal unit problems and ecological bias when developing the methods. In addition, high‐resolution environmental data sets need to be developed, and innovative visualization techniques need to be employed for reporting the outcomes. Implementing these recommendations will not only improve the robustness of scientific findings but also facilitate their translation into practical public health policies and actions.

**Abbreviations**
CVD: cardiovascular disease
INLA: Integrated Nested Laplace Approximation
MAUP: Modifiable Areal Unit Problem
MCMC: Markov‐Chain Monte Carlo
MTUP: Modifiable Temporal Unit Problem
PET: physiologically equivalent temperature
PRISMA‐ScR: Preferred Reporting Items for Systematic reviews and Meta‐Analyses guidelines for scoping review

## Conflict of Interest

The authors declare no conflicts of interest relevant to this study.

## Supporting information

Supporting Information S1

Table S1

Table S2

## Data Availability

The full text PDFs that were retrieved following title and abstract screening cannot be shared directly due to publication copyright and licensing agreements, however the extracted data used for synthesis of this review is made available in the Supporting Information S1.

## Appendix A Search Terms Used in Scoping Review

### A1 Scopus

(TITLE‐ABS‐KEY ( cardiovascular OR “cardiac arrest” OR “heart disease” OR cerebrovascular OR stroke OR cardiorespiratory OR “cardio‐respiratory” OR “cardiopulmonary” OR “cardio‐pulmonary” OR “myocardial infarction” OR “ischaemic heart disease” OR “heart failure” OR cardiomyopathy OR “atrial fibrillation” OR “cerebral infarction” OR atherosclerosis OR embolism OR thrombosis OR “transient ischaemic attack” OR hypertension OR hypertensive OR hypotension OR “coronary artery disease” OR arrhythmia*)

AND TITLE‐ABS‐KEY (environment* OR ambient OR “air quality” OR pollutant OR pollution OR “particulate matter” OR pm2.5 OR pm10 OR nitrogen OR nitrous OR sulphur OR ozone OR carbon OR aerosol OR bushfire OR wildfire OR “wildland fire” OR “forest fire” OR meteorolog* OR temperature OR heat OR cold OR humidity OR greenness OR greenspace OR “green space*” OR “blue space*” OR vegetat* OR “land cover”)

AND TITLE‐ABS‐KEY (spatial OR geospatial OR spatiotemporal OR “spatio‐temporal” OR (small PRE/3 area*)))

### A2 PubMed

(cardiovascular[Title/Abstract] OR “cardiac arrest”[Title/Abstract] OR “heart disease”[Title/Abstract] OR cerebrovascular[Title/Abstract] OR stroke[Title/Abstract] OR cardiorespiratory[Title/Abstract] OR “cardio‐respiratory”[Title/Abstract] OR “cardiopulmonary”[Title/Abstract] OR “cardio‐pulmonary”[Title/Abstract] OR “myocardial infarction”[Title/Abstract] OR “ischaemic heart disease”[Title/Abstract] OR “heart failure”[Title/Abstract] OR cardiomyopathy[Title/Abstract] OR “atrial fibrillation”[Title/Abstract] OR “cerebral infarction”[Title/Abstract] OR atherosclerosis[Title/Abstract] OR embolism[Title/Abstract] OR thrombosis[Title/Abstract] OR “transient ischaemic attack”[Title/Abstract] OR hypertension[Title/Abstract] OR hypertensive[Title/Abstract] OR hypotension[Title/Abstract] OR “coronary artery disease”[Title/Abstract] OR arrhythmia*[Title/Abstract] OR “Cardiovascular Diseases”[MeSH Terms])

AND (environment*[Title/Abstract] OR ambient[Title/Abstract] OR “air quality”[Title/Abstract] OR pollutant[Title/Abstract] OR pollution[Title/Abstract] OR “particulate matter”[Title/Abstract] OR pm2.5[Title/Abstract] OR pm10[Title/Abstract] OR nitrogen[Title/Abstract] OR nitrous[Title/Abstract] OR sulphur[Title/Abstract] OR ozone[Title/Abstract] OR carbon[Title/Abstract] OR aerosol[Title/Abstract] OR bushfire[Title/Abstract] OR wildfire[Title/Abstract] OR “wildland fire”[Title/Abstract] OR “forest fire”[Title/Abstract] OR meteorolog*[Title/Abstract] OR temperature[Title/Abstract] OR heat[Title/Abstract] OR cold[Title/Abstract] OR humidity[Title/Abstract] OR greenness[Title/Abstract] OR greenspace[Title/Abstract] OR “green space*”[Title/Abstract] OR “blue space*”[Title/Abstract] OR vegetat*[Title/Abstract] OR “land cover”[Title/Abstract] OR “Environmental Pollution” OR “Epidemiologic Factors” OR “Meteorological Concepts”[MeSH Terms])

AND (Spatial[Title/Abstract] OR geospatial[Title/Abstract] OR spatiotemporal[Title/Abstract] OR “spatio‐temporal”[Title/Abstract] OR “Spatio‐Temporal Analysis”[MeSH Terms] OR “small areas”[Title/Abstract:∼3])

### A3 Embase

(cardiovascular:ti,ab,kw OR 'cardiac arrest':ti,ab,kw OR 'heart disease':ti,ab,kw OR cerebrovascular:ti,ab,kw OR stroke:ti,ab,kw OR cardiorespiratory:ti,ab,kw OR 'cardio‐respiratory':ti,ab,kw OR 'cardiopulmonary':ti,ab,kw OR 'cardio‐pulmonary':ti,ab,kw OR 'myocardial infarction':ti,ab,kw OR 'ischaemic heart disease':ti,ab,kw OR 'heart failure':ti,ab,kw OR cardiomyopathy:ti,ab,kw OR 'atrial fibrillation':ti,ab,kw OR 'cerebral infarction':ti,ab,kw OR atherosclerosis:ti,ab,kw OR embolism:ti,ab,kw OR thrombosis:ti,ab,kw OR 'transient ischaemic attack':ti,ab,kw OR hypertension:ti,ab,kw OR hypertensive:ti,ab,kw OR hypotension:ti,ab,kw OR 'coronary artery disease' :ti,ab,kw OR arrhythmia*:ti,ab,kw)

AND (environment*:ti,ab,kw OR ambient:ti,ab,kw OR 'air quality':ti,ab,kw OR pollutant:ti,ab,kw OR pollution:ti,ab,kw OR 'particulate matter':ti,ab,kw OR pm2.5:ti,ab,kw OR pm10:ti,ab,kw OR nitrogen:ti,ab,kw OR nitrous:ti,ab,kw OR sulphur:ti,ab,kw OR ozone:ti,ab,kw OR carbon:ti,ab,kw OR aerosol:ti,ab,kw OR bushfire:ti,ab,kw OR wildfire:ti,ab,kw OR 'wildland fire':ti,ab,kw OR 'forest fire':ti,ab,kw OR meteorolog*:ti,ab,kw OR temperature:ti,ab,kw OR heat:ti,ab,kw OR cold:ti,ab,kw OR humidity:ti,ab,kw OR greenness:ti,ab,kw OR greenspace:ti,ab,kw OR 'green space*':ti,ab,kw OR 'blue space*':ti,ab,kw OR vegetat*:ti,ab,kw OR 'land cover':ti,ab,kw)

AND (spatial:ti,ab,kw OR geospatial:ti,ab,kw OR spatiotemporal:ti,ab,kw OR 'spatio‐temporal':ti,ab,kw OR (small NEXT/3 area*))
