# Supplementary material for: Spatiotemporal Approaches to Assess the Association of Environmental Risk Factors With Cardiovascular Diseases: A Scoping Review
Source: Geohealth. 2026 Jan 6;10(1):e2024GH001268. doi: 10.1029/2024GH001268 (PMC12775574; doi:10.1029/2024GH001268)
Supplement: Supplementary file 1 — Supporting Information S1 [file GH2-10-e2024GH001268-s001.docx]

*GeoHealth*

Supporting Information for

**Spatiotemporal approaches to assess the association of environmental risk factors with cardiovascular diseases: a scoping review**

Vishal Singh^1,2^, Susanna Cramb^1,2,3^, Jialu Wang^1^, Wenbiao Hu^1^, Javier Cortes-Ramirez^1,2^

^1^ School of Public Health and Social Work, Queensland University of Technology, Australia

^2^ Centre for Data Science, Queensland University of Technology, Australia

^3^ Australian Centre for Health Services Innovation, Centre for Healthcare Transformation, Queensland University of Technology, Australia

**Contents of this file**

Figure S1: Screening Process (Alternate Version)

**Additional Supporting Information (Files uploaded separately)**

Captions for Table S1: Data Description

Captions for Table S2: Quality Assessment

**Introduction**

This document contains supporting information, including an alternate version of the PRISMA flow diagram, a detailed description of data extracted from the studies included in this review, and the results of quality assessment performed using SMART with rationale for each rating.

**Figure S1.** Screening Process (Alternate Version)

**Additional Supporting Information (Files uploaded separately)**

**Captions for Table S1.** Data Description

File name: “2024GH001268R Table S1 - Data Description.xlsx”

**Captions for Table S2.** Quality Assessment

File name: “2024GH001268R Table S2 - Quality Assessment.xlsx”
